# Appraising perception, accessibility and uptake of DAT among patients with TB

**DOI:** 10.5588/pha.24.0009

**Published:** 2024-06-01

**Authors:** C. Ogbudebe, B. Odume, I. Gordon, O. Chukwuogo, N. Nwokoye, S. Useni, E. Efo, M. Gidado, E. Aniwada, A. Ihesie, D. Nongo, R. Eneogu, O. Chijioke-Akaniro, C. Anyaike

**Affiliations:** ^1^ KNCV Tuberculosis Foundation Nigeria, Abuja, Nigeria;; 2 KNCV TB Plus, The Hague Netherlands,; 3 Department of Community Medicine, University of Nigeria Teaching Hospital, Enugu,; 4 United States Agency for International Development (USAID), Abuja, Nigeria,; 5 National Tuberculosis, Leprosy and Buruli Ulcer Control Programme, Abuja, Nigeria

**Keywords:** tuberculosis, digital adherence technology, Africa

## Abstract

**INTRODUCTION:**

Poor adherence to TB treatment poses a significant public health threat to TB control programmes. The sustainability of directly observed treatment has been questioned because of its non-patient-centred approach and resource-intensive nature, and Digital Adherence Technologies (DATs) provide a suitable alternative. This study assessed the feasibility and acceptability of DATs among patients with TB.

**METHODS:**

This descriptive study was conducted in eight states in Nigeria among all patients with drug-susceptible TB.

**RESULT:**

A total of 230 patients (89.1%) own a phone that no one else uses, and 18 (7.0%) use a family phone. A higher proportion of 189 (73.3%) have airtime credit and 119 (46.1%) have internet credit on their phone. In addition, 216 (83.7%) stated that the reminders they received on their phone helped them remember to take their medicine. Only 11 (4.3%) patients missed a dose of the TB medicine. Equally, 11 (4.3%) patients had taken their TB medicine without using DAT. Of these, 7 (63.3%) did not use DATs because they forgot to text medication labels, and 3 (27.6%) did so because of poor network. Only four (1.6%) purchased additional items to support the use of DATs.

**CONCLUSION:**

DATs are acceptable in a wide variety of settings, even with reported challenges. Implementation efforts should ensure access, address technical challenges, and minimise additional cost to patients.

## Abstract (fr)

**INTRODUCTION:**

La mauvaise observance du traitement antituberculeux constitue une menace importante pour la santé publique pour les programmes de lutte contre la TB. La durabilité du traitement sous observation directe a été remise en question en raison de son approche non centrée sur le patient et de sa nature gourmande en ressources, et les technologies d'observance numérique (DAT) constituent une alternative appropriée. Cette étude a évalué la faisabilité et l'acceptabilité des DAT chez les patients atteints de TB.

**MÉTHODES:**

Cette étude descriptive a été menée dans huit États du Nigeria auprès de tous les patients atteints de TB pharmacosensible.

**RÉSULTAT:**

Un total de 230 patients (89,1%) possèdent un téléphone que personne d'autre n'utilise, et 18 (7,0%) utilisent un téléphone familial. Une proportion plus élevée de 189 (73,3%) ont du crédit de temps d'antenne et 119 (46,1%) ont du crédit Internet sur leur téléphone. De plus, 216 (83,7%) ont déclaré que les rappels qu'ils ont reçus sur leur téléphone les ont aidés à se rappeler de prendre leurs médicaments. Seuls 11 patients (4,3%) ont manqué une dose du médicament antituberculeux. De même, 11 patients (4,3%) avaient pris leur médicament antituberculeux sans utiliser de DAT. De ce nombre, 7 (63,3%) n'ont pas utilisé de fichiers DAT parce qu'ils ont oublié d'envoyer des étiquettes de médicaments par texto, et 3 (27,6%) l'ont fait en raison d'un réseau médiocre. Seulement quatre (1,6%) ont acheté des articles supplémentaires pour soutenir l'utilisation des fichiers DAT.

**CONCLUSION:**

Les fichiers DAT sont acceptables dans une grande variété de contextes, même en cas de problèmes signalés. Les efforts de mise en œuvre doivent garantir l'accès, relever les défis techniques et minimiser les coûts supplémentaires pour les patients.

TB, an infectious disease caused by *Mycobacterium tuberculosis*, is of public health importance and one of the top 10 deadliest infectious diseases worldwide.^1^ Long-term antibiotic treatment lasting for at least 6 months is needed to control TB infection and avoid disease spread.^2^ Based on this, TB treatment is prone to non-adherence, resulting in treatment failure even when effective TB medication is available.^3^ In 2020, approximately 10.0 million people fell ill with TB and an estimated 1.5 million died globally.^4^ It is estimated that in Nigeria, 452,000 (295,000–641,000) persons fell ill with TB in 2020.^4^ At the end of 2019, the treatment coverage rate in Nigeria was 28%, and 71% of patients faced catastrophic costs.^5^

Patient compliance is a key factor in the success of any treatment. Increased evidence has demonstrated that more engaged patients enjoy higher treatment adherence, better health outcomes, and better care experiences compared with those who are less engaged in their care.^6^ Non-compliance with TB treatment poses a significant public health threat, as it is associated with increase in transmission rates, morbidity, and costs to TB control programmes.^7^ It also leads to persistence, as well as resurgence of TB and is regarded as the chief cause of relapse and drug resistance.^8^ It is noteworthy that the interplay of factors determines TB treatment adherence and outcomes, including characteristics of the regimen, attitudes of service providers, and socio-economic, cultural, and environmental factors.^9^

Improving treatment adherence is a key challenge in patients with TB. The sustainability of directly observed treatment (DOT) has been questioned because of its non-patient-centred approach and resource-intensive nature.^10,11^ As such, in many high TB burden settings, 15% or more of patients with TB do not complete treatment. This consequently can adversely affect the health and economic wellbeing of patients, caregivers, and health systems.^12,13^

The WHO has ensured adherence as a principal component of global ‘End TB Strategy’.^13^ In furtherance to this, in April 2017, the WHO in its comprehensive update on guidelines for drug-susceptible TB treatment advised the use of digital adherence technologies (DATs) to improve adherence with evidence-based recommendations on the use of electronic medication monitors to help patients adhere to TB medication.^14^ Critical to the successful use of such devices in improving patient medication adherence is the ability of providers and patients to effectively use the technology. To design and deploy an electronic monitoring device optimally, one must consider patient and provider acceptability, satisfaction, and feasibility.^14^

A recent real world randomised trial in Uganda involving DOT vs. DAT demonstrated that 99DOTS can be iteratively adapted to meet local user needs, effectively facilitate treatment adherence, and improve treatment success.^15^ 99DOTS has been promoted as a low-cost, scalable intervention to help patients with TB adhere to treatment.^15^ Although it is expected that SMS, telephone calls and video directly observed therapy (VDOT) may replace in-person DOT, patients' ability to participate in these programmes depends on the patients place of residence, telecommunication infrastructure, phone availability and connection costs.^14^

DATs are increasingly being implemented as an alternative to traditional DOT for TB treatment.^16^ A study noted that TB patients acknowledged that SMS could serve as medication reminders to overcome forgetfulness and offers them the opportunity to familiarise oneself with medication adherence, especially among those who did not have experience taking medication regularly.^17^ However, to date there are limited data on their feasibility and acceptability among persons on treatment which this study aims to assess.^16^

## MATERIALS AND METHODS

### Study area and setting

The study was carried out in 8 states in Nigeria, 4 in the North region (Benue, Kaduna, Kano and Nasarawa states) and 4 in the South regions (Akwa Ibom, Anambra, Imo and Rivers). These states combined show a treatment success rate of 90% and reflect a mix of TB burden and gaps in treatment success rate within the 14 states where KNCV Nigeria implements the TB LON Regions 1&2 Project. KNCV Nigeria, in collaboration with her technical partners, implemented 99DOTS and video-observed therapy (VOT) in 98 health facilities that provide DOTS across eight states. Patients enrolled on 99DOTS were required to text a unique hidden code on their medication labels to the country short-code 3340 as proxy confirmation of medication intake while VOT patients sent daily videos using a smart phone. Patients who failed to send videos or text messages were sent reminder messages by 6 pm daily and all adherence data were made available to the healthcare workers (HCWs) on an adherence platform for patient management.

### Study population

All drug-susceptible TB patients on treatment at facilities in the eight (8) select states during the study period, who consent to participate in the DAT study and are enrolled on DAT at the start of their treatment. However, patients transferred to/from another facility to complete treatment and patients who already started treatment before enrolment on DAT were excluded.

### Study design and duration

The study was a descriptive cross-sectional study using a questionnaire to assess patients’ perception of accessibility and uptake of DATs among selected facilities. The study lasted for 3 months (October–December 2022)

### Sample size determination and sampling techniques

All TB patients that presented in the health facilities in selected states were studied.

### Data collection

Data was collected using a semi-structured questionnaire involving open-ended questions about the characteristics of patients, ownership of phones, access to network services, opinion on and acceptability of DATs.

### Data analysis and presentation

Data was analysed using IBM SPSS v25 (IBM, Armonk, NY, USA). Categorical variables were summarised using proportion and percentages. They were presented in tables.

### Ethical consideration

Ethical clearance was received from the National Health Research Ethics Committee (NHREC), Federal Ministry of Health, Abuja, Nigeria. The project obtained signed informed consent from patients and only non-identifying patient data were collected as part of the study. Voluntary participation and confidentiality were ensured.

## RESULT

Table 1 shows characteristics of patients. The mean of age the respondents was 34.63 +/- 14.81 years. A higher proportion were males (*n* = 161, 62.4%), single (*n* = 129, 50.0%), had secondary education (*n* = 128, 49.6%), self-employed (*n* = 123, 47.7%), had 1–3 adults (age: 18+ years) living with them (*n* = 136, 52.8%), and had no child aged 0–5 years living with them (*n* = 164, 63.6%).

**TABLE 1.** Characteristics of patients.

Variables	Frequency
	(*n* = 258)
	*n* (%)
Sex	
Female	97 (37.6)
Male	161 (62.4)
Age categories, years	
<18	22 (8.5)
18–32	105 (40.7)
33–47	80 (31.0)
>47	51 (19.8)
Mean ± SD	34.63 ± 14.81
Marital status	
Married	121 (46.9)
Single	129 (50.0)
Others	8 (3.1)
Educational level	
Non-formal education	17 (6.6)
Primary	49 (19.0)
Secondary	128 (49.6)
Tertiary	64 (24.8)
Current occupation	
Civil servant	21 (8.1)
Not employed	60 (23.3)
Private organisation	21 (8.2)
Self-employed	123 (47.7)
Student	33 (12.8)
Adults (18+ years) you currently live with	
0	53 (20.5)
1–3	136 (52.8)
>3	69 (26.7)
Children (6–17 years) you currently live with	
0	121 (46.9)
1–3	89 (34.6)
>3	48 (18.5)
Children (0–5 years) you currently live with	
0	164 (63.6)
1–3	82 (31.9)
>3	12 (4.8)

SD = standard deviation.

Table 2 shows ownership, access and use of mobile phone of patients as well as ancillary expenses incurred in course of treatment for patients. The majority of them (*n* = 230, 89.1%) owned a phone that no one else used, while 18 (7.0%) used family phone of which they were not the primary owners. A higher proportion (*n* = 189, 73.3%) had airtime credit on their phone sometimes and 66 (25.6%) always. Moreover, a higher proportion (*n* = 119, 46.1%) had internet credit on their phone sometimes, and 41 (15.9%) always. Only 4 (1.6%) bought additional items to support the use of DAT. Items bought were phone credit/airtime (*n* = 3, 75.0%) and data or internet package (*n* = 1, 25.0%). Of these, 2 (50.0%) spend 500 Nigerian naira (NGN; equivalent to USD0.78) and 1 (25.0%) spent NGN 200 (USD0.31) and NGN 1,500 (USD2.35) each.

**TABLE 2.** Ownership, access and use of mobile phone and ancillary expenses incurred in the course of treatment.

Variables	Frequency
	(*n* = 258)
	*n* (%)
Have access to or own a mobile phone	
Yes, I own a phone that no one else uses	230 (89.1)
Yes, family shares a phone - I am the primary owner	10 (3.9)
Yes, family shares a phone - I am NOT the primary owner	18 (7.0)
Frequency of having airtime credit on your phone	
Always	66 (25.6)
Never	2 (0.8)
Not applicable	1 (0.4)
Sometimes	189 (73.3)
Frequency of having mobile internet credit on your phone	
Always	41 (15.9)
Never	10 (3.9)
Not applicable	88 (34.1)
Sometimes	119 (46.1)
Since starting TB treatment, ever bought additional items to support the use of DAT	
No response	180 (69.8)
No	74 (28.7)
Yes	4 (1.6)
Items you bought with your own money (*n* = 4)	
Phone credit/airtime	3 (75.0)
Changes for data or internet package	1 (25.0)
How much you paid for these items (*n* = 4)	
200	1 (25.0)
500	2 (50.0)
1,500	1 (25.0)

DAT = digital adherence technology.

Table 3 shows opinion on digital adherence technology (DAT) for patients. Out of all patients, 228 (88.3%) knew how to use DAT, 227 (88.0%) will recommend using DAT to their family or friends if they have TB, 220 (85.3%) agreed that the instructions on the DAT makes it easy for them to remember what to do, 216 (83.7%) stated that the reminders they receive on their phone help them to remember to take their medicine and 215 (83.3%) use DAT while taking TB medicines will help them get healthy. A higher proportion were equally positive on other opinion on DAT (all at least 62.5% and above). However, 64 (24.8%) said that it takes them too much time to record themselves taking their TB medicine, or text 3340 for the labels every day and 90 (34.9%) were worried that using the DAT will make them more likely that others will find out that they have TB.

**TABLE 3.** Opinion on DAT.

Variables	Positive	Negative
	*n* (%)	*n* (%)
Opinion on DAT		
I know how to use the DAT	228 (88.3)	30 (11.7)
I am more connected to my healthcare provider while using my DAT	213 (82.5)	45 (17.5)
I make fewer trips to the health clinic for my TB treatment because I am using an DAT	211 (81.8)	47 (18.2)
I am comfortable using my DAT in front of other people.	199 (77.1)	59 (22.9)
I am worried that using the DAT will make it more likely that others will find out that I have TB	90 (34.9)	168 (65.1)
It takes me too much time to record myself taking my TB medicine, or text 3340 for the labels every day	64 (24.8)	194 (75.2)
Using my DAT will help me complete my TB treatment	214 (83.0)	44 (17.0)
The reminders I receive on my phone help me to remember to take my medicine	216 (83.7)	42 (16.3)
Using DAT while taking TB medicines will help me get healthy	215 (83.3)	43 (16.7)
I will recommend using DAT to my family or friends if they have TB	227 (88.0)	31 (12.0)
The instruction on the DAT makes it easy for me to remember what to do	220 (85.3)	38 (14.7)
I am comfortable using the DAT outside of my home	205 (79.4)	53 (20.6)
I am concerned about the privacy of my health information collected by the DAT	194 (75.2)	64 (24.8)
If you could change anything about the adherence technology, what you would change		
Make me not have to send a text to a number	5 (1.9)	253 (98.1)
Increase the frequency of reminders	82 (31.8)	176 (68.2)
Decrease the frequency of reminders	3 (1.2)	255 (98.8)
I would use it for all my medications	13 (5.0)	245 (95.0)
I would not use VOT/medication labels at all	1 (0.4)	257 (99.6)
Visual appearance of the medication labels	2 (0.8)	256 (99.2)
I would not change anything	143 (55.4)	115 (44.6)

DAT = digital adherence technology; VOT = video-observed therapy.

When asked if they could change anything about the adherence technology, what they would change, about 143 (55.4%) would not change anything, 82 (31.8%) wanted to increase the frequency of reminders and 13 (5.0%) would use DAT for all their medications. Table 4 shows TB medication activities of patients and health workers’ assistance/involvement for patients. Out of all patients, 180 (69.8%) use a mix of methods, 29 (11.2%) do not require reminder and 21 (8.1%) were from family member or friends. A higher proportion spends less than 1 minute to send a text to the number [3340] for those that use medication labels. Only 37 (14.3%) have been shown what their adherence information looks like (e.g., how many doses you have taken) by HCW. Those that responded to calls from health workers spent few minutes on the call. The majority have not been visited by health worker.

**TABLE 4.** TB medication activities of patients and health workers’ assistance/involvement.

Variables	Frequency
	(*n* = 258)
	*n* (%)
Ways you remember to take your TB medications	
Mix of all the options	180 (69.8)
A family member or friend reminds me	21 (8.1)
I get an SMS message	19 (7.4)
I set an alarm (on phone or other device)	9 (3.5)
Do not require a reminder	29 (11.2)
If using the medication labels, how much time does it take for you to send a text to the number [3340]	
No response	182 (70.5)
1–2 min	16 (6.2)
3–5 min	4 (1.6)
≥6 min	1 (0.4)
<1 min	49 (19.0)
Not applicable	6 (2.3)
Has a healthcare worker ever shown you what your adherence information looks like (e.g., how many doses you have taken)	
No response	180 (69.8)
No	41 (15.9)
Yes	37 (14.3)
On average, how many minutes did you spend on the call with the healthcare worker when you called	
No response	227 (88.0)
1–5 min	18 (7.0)
<1 min	4 (1.6)
>5 min	9 (3.5)
In the last 2 months, the number of times a healthcare worker visited you at home/work about your TB treatment	
0	70 (27.1)
1	3 (1.2)
3	2 (0.8)
4	3 (1.2)

Table 5 displays the utilisation of adherence technology by patients, instances of missed doses of TB medication, and the responses of health workers to these occurrences. Only 11 individuals (4.3%) had ever taken their TB medicine without utilizing Directly Observed Treatment (DAT). Among these, 7 (63.3%) did not use DAT because they forgot to text medication labels, 3 (27.6%) due to poor network connection, and 1 (9.1%) because they lacked access to a phone. Approximately 63 patients (24.4%) mentioned that people they lived with were aware of their use of DAT for TB treatment, while 15 (5.8%) affirmed that nobody knew. Additionally, only 11 patients (4.3%) had ever missed a dose of TB medicine. Among them, 4 (36.3%) missed 1 dose, 2 (18.2%) missed 3 doses, and 3 (27.3%) missed 5 doses. The primary reasons for missing doses were forgetfulness (*n* = 8, 72.7%), side effects (*n* = 2, 18.2%), and not wanting someone to observe them taking the drugs (*n* = 1, 9.1%). Following a missed dose of TB medicine, 4 patients (36.3%) were not contacted by a health worker, while 5 (45.5%) were contacted the next day, and 2 (18.2%) were contacted 2 days or more later.

**TABLE 5.** DAT use: missed doses of TB medicine and healthcare worker responses.

Variables	Frequency
	(*n* = 258)
	*n* (%)
Ever taken your TB medicine without using DAT	
No response	180 (69.8)
No (0 times)	67 (26.0)
Yes, a few times (1–3 times)	2 (0.8)
Yes, many times (≥4 times)	9 (3.5)
Reasons for not using the DAT (*n* = 11)	
Did not have access to a phone	1 (9.1)
Network connection was poor	3 (27.6)
I forgot to text medication labels	7 (63.3)
People you live with know that you use DAT for TB treatment	
No response	180 (69.8)
No	15 (5.8)
Yes	63 (24.4)
Number of doses of your TB medicine missed in the last 30 days (*n* = 11)	
1	4 (36.3)
2	1 (9.1)
3	2 (18.2)
4	1 (9.1)
5	3 (27.3)
Reasons for missing the doses (*n* = 11)	
Side effects	2 (18.2)
Forgot to take	8 (72.7)
Did not want anyone to see me taking the medicines	1 (9.1)
Following missed a dose of your TB medicine, when your healthcare worker contacted you (*n* = 11)	
The healthcare worker did not contact me	4 (36.3)
The next day	5 (45.5)
≥2 days	2 (18.2)

## DISCUSSION

Findings from this study show that the majority of patients owned a phone that no one else uses. All participants were satisfied with use of DATs. Only a few (4.3%) had ever taken their TB medicine without using DAT. Of these, 63.3% did not use DAT because they forgot to text medication labels and 27.6% due to network connection being poor. Limited numbers (4.3%) missed doses of TB medicine with the range of missed doses being one to five. Reason for missing the doses were majorly that they forgot to take the drugs followed by side effect and few not wanting someone to know that they take the drugs. This is expected as TB is commoner among persons of low socio-economic status. Also, network issues are common in the country. Research has previously alluded that although cellular infrastructure and mobile phone ownership are advancing globally, poor network coverage, low-quality phones, and shared phone usage remain as limitations. Importantly, a study in Peru showed that mobile phone access is lowest among the poorest patients with TB.^18^

Other earlier documented reasons for missed videos were phone malfunction, uncharged battery and app malfunctions.^19^ The same study noted that 92% of patients reported being very satisfied with using VDOT.^19^ In another study, people with TB had an overall positive impression of DATs with pooled estimates between 4.0 to 4.8 out of five across categories. However, 44% of people with TB reported taking TB medications without reporting dosing via DATs and 23% reported missing a dose of medication.^20^ Common reasons identified in the study included problems with electricity, network coverage, and technical issues with the DAT platform. DATs were overall perceived to reduce visits to clinics, decrease cost, increase social support, and decrease workload of HCWs.^20^

Prior study found DATs to be technically feasible nevertheless identified potential challenges to include; the impact of the technology on confidentiality, shared phone ownership, usability skills, and availability of electricity.^21^ Decreasing adherence has also been reported due to side effects^22^ and indifference by patients in engaging with the DAT to record the dose they have taken.^23^ Worthy of note is that this might not necessarily reflect suboptimal medication intake.^24^ For example, a study on whether or not the use of 99DOTS accurately represents if a dose was taken found that 99DOTS tends to underestimate adherence.^25^

Most participants in current study stated that the reminders they receive on their phone help them to remember to take their medicine even though that a few said that it takes them too much time to record themselves taking their TB medicine, or text 3340 for the labels every day. Also, a sizeable percentage were worried that using the DAT will make them more likely that others will find out that they have TB. This is supported by earlier studies. A study in southwestern Uganda, participants recounted that DAT are generally acceptable with the major feedback being perceived utility that intervention was beneficial in motivating and reminding patients to take medication, as well as enabling provision of social support.^20^ This implies that mobile telephones could provide alternative approaches to providing social support for TB medication adherence especially where patients do not stay close to their social supporters.

Although there is an increasing use of DATs, such as real-time monitors and SMS reminders, in TB medication adherence, research to date reports mixed results.^16^ Differences in findings may relate to the extent of patient involvement in technology development, context, culture, and technology exposure. Additionally, despite their potential, data supporting the impact of DATs on treatment success and mortality are limited,^26,27^ and suboptimal patient engagement with various DATs for TB has been reported.^28,29^

Previous studies in high-income^30^ and low-income countries^31,32^ have reported high adherence and patient satisfaction with VDOT. Equally, VDOT can overcome the limitations of in-person DOT at the patient and health system levels. For example, VDOT study has shown that the distance barrier is mostly eliminated,^33^ patients have greater autonomy to choose when and where to take their medications,^33^ the costs of travel are minimised and providers can support a higher number of patients, thus increasing the health system efficiency.^33^

An insignificant number of participants bought additional items to support the use of DAT. This should be avoided as it may hamper uptake of DATs bearing in mind that the majority of the patients are already poor. This is supported by another study that reported financial constraints as a notable limitation and proposed a potential role for financial incentives.^34^

## CONCLUSION

The majority of the patients own a phone, have airtime credit for internet on their phone sometimes and were satisfied with use of DATs. Almost all comply by taking their TB medicine using adherence technology and, on few occasions, did not send messages as they forgot or due to poor network connection. Limited numbers missed doses of TB with major reason because they forgot to take the drugs followed by side effect. This is in line with other previous studies. Although DATs were acceptable in a wide variety of settings, there were challenges related to the feasibility of using current DAT platforms. Implementation efforts should concentrate on ensuring access, anticipating, and addressing technical challenges, and minimising additional cost to people with TB.
